# Speed limits and their effect on air pollution in Mexico City: A quasi-experimental study

**DOI:** 10.1016/j.scitotenv.2024.171506

**Published:** 2024-05-10

**Authors:** Jose Luis Texcalac-Sangrador, Carolina Pérez-Ferrer, Carolina Quintero, Francisco-Javier Prado Galbarro, Goro Yamada, Nelson Gouveia, Tonatiuh Barrientos-Gutierrez

**Affiliations:** a Center for Research in Population Health, National Institute of Public Health, Cuernavaca, Morelos, Mexico; b Department of Research, Hospital Infantil de México Federico Gómez, Mexico City, Mexico; c Urban Health Collaborative, Dornsife School of Public Health, Drexel University, Philadelphia, PA, USA; d Department of Preventive Medicine, University of Sao Paulo Medical School, Sao Paulo, Brazil

**Keywords:** Speed, Road safety, Air pollution, City, Mexico, Policy

## Abstract

Speed limits are an evidence-based intervention to prevent traffic collisions and deaths, yet their impact on air pollution in cities is understudied. The objective of this study was to investigate the association between lower speed limits and air pollution. We leverage the introduction of a new road safety policy in Mexico City in December 2015 which lowered speed limits, increased fines, and installed speed radars to enforce compliance. We tested whether the policy had an impact on particulate matter (PM_2.5_) and nitrogen dioxide (NO_2_) at the city level, and whether air-quality monitoring stations' proximity to speed radars moderated this effect due to more acceleration and deceleration around radars. NO_2_ and PM_2.5_ concentrations from January 2014 to December 2018 were obtained from the National System of Air Quality Information. Air-quality monitoring stations were classified as in close-proximity or far-from-speed radars. Interrupted time series analyses were conducted for each outcome separately, using linear mixed models and adjusting for seasonality and time-varying confounders: registered vehicles, temperature, wind-speed and relative humidity. The results suggest improvement in both contaminants after the speed limits policy. For NO_2_, the pre-policy trend was flat, while the post-policy trend showed a decline in concentrations of 0.04 ppb/week. For PM_2.5_, concentrations were increasing pre-policy by 0.08 μg/m^3^ per week, then this trend flattened in the post-policy period to a weekly, non-significant, increase of 0.03 μg/m^3^ (*p* = 0.08). Air-quality monitors' proximity to speed radars did not moderate the effect of the policy on either of the pollutants. In conclusion, the speed limits policy implemented in Mexico City in 2015 was associated with improvements in air pollution.

## Highlights

• Speed limits are recommended for road safety but may impact air pollution.
• We evaluated a new speed limits policy implemented in Mexico City.
• NO_2_ and PM_2.5_ concentrations improved after speed limits policy was implemented.
• Air quality monitors' proximity to speed radars did not modify NO_2_ or PM_2.5_ readings.

## Graphical abstract

**Figure:**
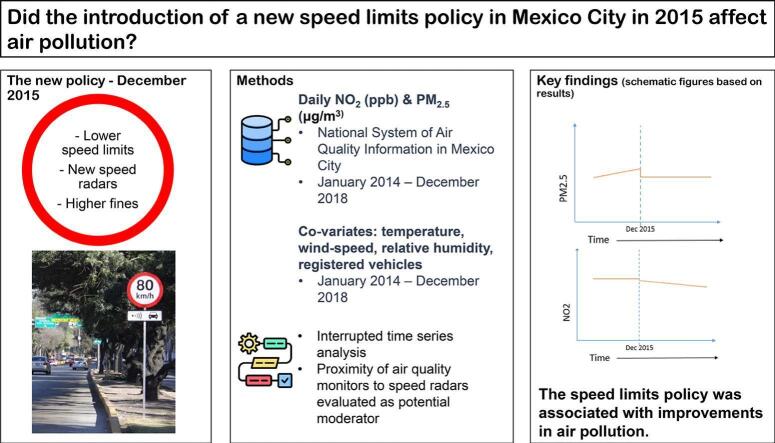

## 1 Introduction

Speed limits are an evidence based, widely used policy to prevent motor vehicle collisions and deaths (Sadeghi-Bazargani and Saadati, 2016). Yet, their environmental impact, especially in cities, is controversial (Bigazzi and Rouleau, 2017; Folgerø et al., 2020). In urban highways, lowering speeds may result in lower vehicle emissions and traffic related air pollution because nitrogen oxide (NOx) emissions increase at higher speeds (above 100 km/h) due to higher combustion temperatures (Keuken et al., 2010; Lopez-Aparicio et al., 2020). However, in secondary roads of urban areas, such as residential streets and avenues, there may be an adverse effect of lower speeds on air pollution if they increase trip duration, density of vehicles in the road network and acceleration and deceleration (Coelho et al., 2005; Gedik et al., 2022; Int Panis et al., 2006). Further, exhaust particulate matter (PM) emissions are highest at low speeds (below 40 km/h) due to incomplete combustion (Keuken et al., 2010).

Based on the evidence of lower speeds leading to fewer emissions, speed limit policies have been used by some European cities to improve local air quality, particularly on urban highways (Folgerø et al., 2020). Studies evaluating their effect on vehicle emissions and traffic related air pollution using measured data provide mixed results. In Oslo, reducing the speed limit from 80 km/h to 60 km/h had no effect on nitrogen dioxide (NO_2_), NOx or particulate matter (PM_2.5_ or PM_10_) (Folgerø et al., 2020). Similarly, in Barcelona, reductions in the speed limit from 100 km/h or 120 km/h to 80 km/h did not have an effect on NOx or particulate matter (Bel et al., 2014), but in Amsterdam, a reduction in the speed limit from 100 km/h to 80 km/h with strict enforcement led to a reduction in PM_10_ and NOx (Dijkema et al., 2008; Keuken et al., 2010).

On the other hand, at lower speeds which are more typical in cities, and around speed monitoring devices, lowering speed limits may lead to increased emissions and air pollution. A modelling study of traffic across a hypothetical road network concluded that the effect of reduced average speed on emissions is offset by frequent acceleration and deceleration movements (Int Panis et al., 2006). In Portugal, traffic signals intended to warn drivers of exceeding speed limits, led to increases in carbon monoxide (CO), NO and hydrocarbon (HC) emissions of between 15 and 40 % (Coelho et al., 2005). Increases in emissions may occur due to delays, queue formation and speed change cycles for approaching traffic (Coelho et al., 2005).

In 2015, Mexico City adopted new road traffic regulations motivated by high road traffic mortality. The new policy lowered speed limits and increased fines for speeding vehicles (Gaceta Oficial del Distrito Federal, 2015). The new speed limits were: 80 km/h in freeways with grade-separated road junctions (new speed limit), 50 km/h in arterials (previously 70 km/h), 40 km/h in collectors (no change), and 20 km/h in school zones (no change). To enforce speed limits, the City government installed 25 new automated speed enforcement devices. These came in addition to 18 existing devices that were installed between 2012 and 2014. Detection of speeding by these devices led to license plate recognition and automatic fining. The policy was approved on August 17, 2015 and came into effect on December 15, 2015.

The new policy was met with skepticism by some environmental groups that claimed that it would lead to increased air pollution due to longer trip duration and congestion (Mochan, 2016; Olivares Alonso, 2016). To date, the policy's effect on road safety has been studied, (Quintero Valverde et al., 2022) but its's effect on air pollution has not. Further, the policy provides a good natural experiment to investigate lower speeds in urban areas using measured data. As related above, previous studies that use measured data have focused on urban highways with high speeds. To address the paucity of data on this issue, this study aims to investigate the association between lower speed limits and air pollution by leveraging the introduction of the new road safety policy in Mexico City in 2015 (Gaceta Oficial del Distrito Federal, 2015).

Specifically, we aimed to answer the following questions, did the 2015 introduction of lower speed limits in Mexico City have an effect on air pollution, and was the effect (if any) moderated by air quality monitors' proximity to speed radars? We hypothesized that a station in close proximity to radars may have higher readings of the contaminants of interest compared to monitoring stations that were far-from-radars because drivers may decrease their speed upon seeing a speed camera and then accelerate when it is behind them, producing more emissions. By conducting this analysis, we will be able to ascertain the potential environmental impacts of speed limits, facilitating future risk-benefit analyses of speed limit programs.

## 2 Materials and methods

### 2.1 Data sources

#### 2.1.1 Outcome variables: NO_2_ and PM_2.5_

Hourly NO_2_ and PM_2.5_ concentrations from January 2014 to December 2018 were obtained from automatic monitoring stations of the National System of Air Quality Information (SINAICA by its acronym in Spanish) (Secretaría de Medio Ambiente de la Ciudad de México, 2016). SINAICA is the government agency in charge of gathering all national air quality monitoring information and evaluating compliance with Mexican air quality standards (Secretaría de Gobernación, 2021). These standards follow strict information quality criteria, considering the recommendations and methods of the WHO and EPA air quality guidelines.

Data from all fixed-location monitoring stations measuring PM_2.5_ or NO_2_ located in Mexico City were included; 16 stations for PM_2.5_ and 15 stations for NO_2_.

For each included monitoring station and for each pollutant (NO_2_ and PM_2.5_) we calculated daily average concentration of non-peak traffic hours. A missing value for the day was entered if data was not available for at least 75 % of the non-peak hours of the day. Supplementary Figs. 1 and 2 detail data missingness per day for each contaminant. Data on NO_2_ was available for 78.1 % of days, while data for PM_2.5_ was available for 60.5 % of days. Missing data occurs when a station loses power or is temporarily damaged or out of service. Missingness is not related to air pollution readings nor with the speed limits policy, therefore we believe it does not bias our main analysis. We restricted air pollution data collection to daily non-peak hours (from 9 am to 12 pm, 3 pm to 6 pm and 7 pm to 9 pm, 8 h total (Instituto Nacional de Estadística y Geografía, 2017)) because these are the periods when drivers must observe speed limits and radars because traffic is flowing. The holidays were also excluded because of atypical driving behavior during those times.

#### 2.1.2 Covariates

Data for temperature, relative humidity and wind speed were obtained from SINAICA and were available on an hourly basis for each monitoring station. As with pollutants, we estimated daily averages from non-peak hours. Considering these time-varying variables in the analysis is important because relative humidity and wind speed are associated with coarse and fine road dust resuspension which affects particulate matter concentrations (Matthaios et al., 2022). Temperature also affects pollutant concentration and dispersion (Bel et al., 2014). When meteorological data from a monitoring station was not available, the value was interpolated by inverse distance weighting (IDW).

The number of registered vehicles in Mexico City were obtained on a monthly basis for the period of analysis from the National Institute of Geography and Statistics (INEGI by its acronym in Spanish) (Instituto Nacional de Geografía y Estadística, 2019). This variable was used as a proxy for traffic volume and is expected to be related to the concentration of pollutants, given that traffic exhaust and non-exhaust emissions are their main source (Matthaios et al., 2022).

### 2.2 Classification of air quality monitors

**Fig. 1:**
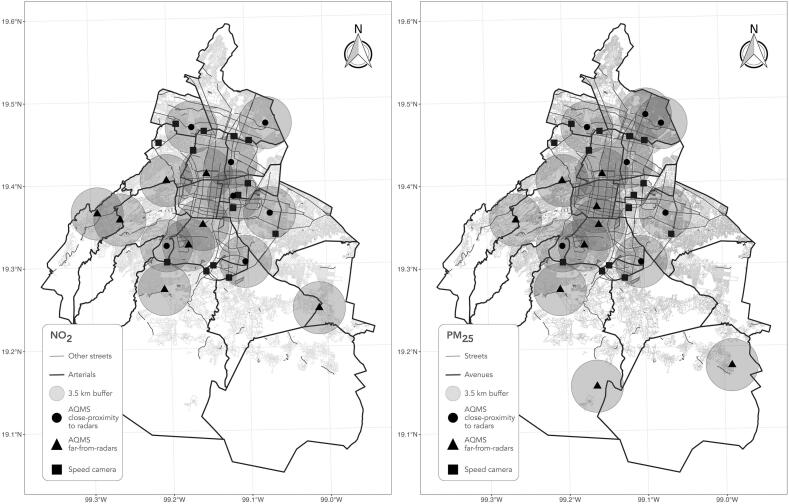
Location of air quality monitoring stations in Mexico City and their classification according to proximity to speed radars.

Monitoring stations were georeferenced based on their geographic coordinates (Fig. 1) and a 3.5-km buffer was generated around each one of them. Similarly, speed radars were georeferenced. Then, the number of speed radars within each monitor's buffer was quantified. Monitoring stations were classified as in close-proximity to radars or far-from-radars according to the presence or absence of radars inside their buffers (Fig. 1). The classification of the monitors was carried out considering that in the presence of a radar, drivers decrease their speed and cause greater congestion, consequently a station in close proximity to radars may have higher readings of the contaminants of interest compared to monitoring stations that are far-from-radars. We tested different size buffers but made the pragmatic decision to keep 3.5 km buffers to better classify stations. Smaller buffers led to very few monitors in ‘close-proximity’ to speed radars.

The total number of stations meeting inclusion criteria for NO_2_ was 15, of which 7 had a speed radar within its buffer (close-proximity), while for PM_2.5_ a total of 16 stations were included, of which 7 had a speed radar within its buffer (close-proximity) (Fig. 1).

### 2.3 Statistical analyses

We first analyzed the effect of the speed limits policy on air pollution at the city level with an interrupted time series (ITS) analysis for each outcome separately.(Bernal et al., 2017) There are three main variables in each model: the outcome (Y), daily NO_2_ or PM_2.5_, the time elapsed since the start of the study in weeks (T) and a dummy variable indicating the policy coded 0 before the policy was implemented and 1 after the policy implementation (Xt).

The following basic model was tested:

(1) $$\;Y_{t}=\beta_{0}+\beta_{1}\left(T-T_{i}\right)+\beta_{2}X_{t}+\beta_{3}\;\left(T-T_{i}\right)X_{t}$$

where Yt is the outcome at time t, β0 represents the pollutant level at the start of the intervention $\left(T-T_{i}\right)$ = 0, β1 is interpreted as the weekly change in the outcome before policy implementation (representing the underlying pre-policy trend), β2 is the change in pollutant level immediately after the 2015 policy implementation, and β3 indicates the slope change following the 2015 policy (using the interaction between time centered at the time of the policy, $T-T_{i},$and policy $X_{t}$) (Xiao et al., 2020). The pre-policy period was defined from 1st January 2014 to 14 December 2015, while the post-policy period was defined as 15 December 2015 to 31st December 2018. The time series stops in December 2018 because the speed limits policy was relaxed with a new government taking office in January 2019 (Quintero Valverde et al., 2022).

As a second step we conducted a controlled interrupted time series analysis (CITS) (Bhaskaran et al., 2013; Lopez Bernal et al., 2018a) to investigate whether air quality monitor's proximity to speed radars modified the effect of the 2015 speed limits policy. We compared the time series for each outcome for two groups: close-proximity group, defined as monitors with at least one speed camera within a 3.5 km buffer and the far-from-radars group, defined as monitors without speed cameras within their 3.5 km buffers. In addition to the three variables described in Eq. (1), we introduce G which denotes monitors in close proximity to radars (G = 1) or far from radars (G = 0) and the interaction GX$\left(T-T_{i}\right)$, which represents the difference in the time slope following the start of the policy in close-proximity compared to far-from-radars monitors. See Eq. (2).

(2) $$Y_{t}=\beta_{0}+\beta_{1}\left(T-T_{i}\right)+\beta_{2}X_{t}+\beta_{3}\left(T-T_{i}\right)X_{t}+\beta_{4}G+\beta_{5}G\left(T-T_{i}\right)+\beta_{6}GX_{t}+\beta_{7}GX_{t}\left(T-T_{i}\right)$$

We tested for both a change in the intercept and in the slope of the close-proximity and far-from-radar time series, to capture the immediate impact of speed limits and the longer-lasting effects that should occur as more drivers become aware of speed radars and fines. Therefore, $\beta_{6}$ represents the difference between the change in intercept in the close-proximity and far-from-radar groups associated with the policy (new speed limits) and $\beta_{7}$ represents the difference between the change in slope in the close-proximity and far-from-radar groups associated with the policy. These two parameters are the measures of effect.

As a sensitivity analysis we repeated the city level analysis moving the start date of the policy to 15 June 2016 (i.e., a 6-month lag). The rationale for this analysis is that the effect of the speed limits policy on driver behavior and air pollution may have not been immediate. It may have taken drivers some time to realize they were being fined for exceeding the new speed limits and this may have delayed adoption of lower speeds.

Both the ITS and CITS analyses were conducted as described above for each outcome separately (PM_2.5_ and NO_2_) using generalized linear mixed models considering a hierarchical data structure where pollutant concentrations over time were nested within monitoring stations. Models included a random intercept for air quality monitoring stations and a random slope for time and were adjusted for the following time varying confounders: temperature, wind speed, relative humidity and number of registered vehicles for Mexico City (Quintero Valverde et al., 2022). All covariates were centered at their mean. Further, models were adjusted for seasonality patterns in the data using Fourier terms (Bernal et al., 2017) and we applied robust variance estimators to account for correlations that might not be correctly accounted for in our models. ITS studies are not affected by variables that remain fairly constant over time because these are taken into account when modelling the underlying long-term trend (Bernal et al., 2017). Data processing was conducted in R version 4.1.0 while analyses were conducted in STATA 14.

## 3 Results

**Fig. 2:**
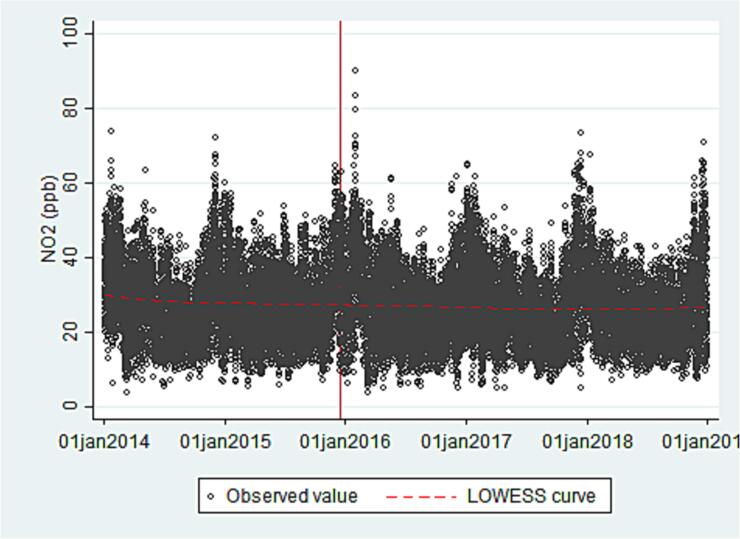
Daily non-peak-hour NO_2_ concentrations, measured by 15 stations in Mexico City. January 2014 to December 2018. *the red vertical line represents the start of the speed limits policy.

**Fig. 3:**
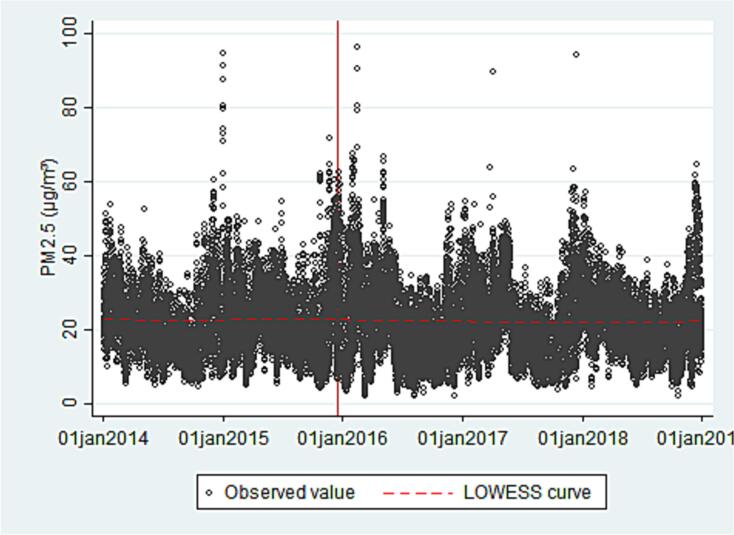
Daily non-peak-hour PM_2.5_ concentrations, measured by 16 stations in Mexico City. January 2014 to December 2018. *the red vertical line represents the start of the speed limits policy

The total number of observations was 7988 (daily concentration averages) pre-policy and 12,950 post-policy for NO_2_ and 6646 (daily concentration averages) pre-policy and 10,869 post-policy for PM_2.5_. Table 1 shows the mean concentration of NO_2_ and PM_2.5_ in the pre- and post-policy periods, as well as the mean value of all covariates. Very small differences can be observed across the periods with the exception of the number of registered vehicles which increased. Fig. 2, Fig. 3 show the trends in NO_2_ and PM_2.5_ over time; the vertical lines mark the start of the speed limits policy. For both contaminants, seasonal fluctuations can be observed, with highest concentrations in the winter months.

**Table 1:** Pre and post policy averages for outcome variables and covariates across all monitors.

	Pre-2015 (Jan 2, 2014 - Dec 14, 2015)			Post-2015 (Dec 15, 2015–31 Dec 2018)		
	Mean	sd	Min, max	Mean	sd	Min, max
NO_2_ (ppb)	27.6	9.5	3.9, 73.5	26.6	9.6	3.9, 89.9
PM_2.5_ (μg/m^3^)	22.6	9.4	4.1, 94.5	22.3	9.9	2.1, 96.1
Temperature (°C)	16.7	2.3	7.5, 23.0	16.6	3.1	2.1, 24.8
Relative humidity (%)	58.0	13.4	15.7, 94.5	55.2	14.8	10.7, 98.0
Wind speed (m/s)	2.0	0.4	0.9, 4.9	2.1	0.6	0.8, 8.0
Registered vehicles⁎	479.9	11.5	463.0, 499.8	534.4	26.9	496.8, 580.1

⁎ In 10,000 s

Table 2 shows the results of the interrupted time series analyses for PM_2.5_ and NO_2_ at the city level. This analysis suggests that there was a decline in both contaminants after the speed limits policy. For NO_2_, the pre-policy trend was flat, while the post-policy trend shows a weekly decline in concentrations of 0.04 ppb. For PM_2.5_, concentrations were increasing pre-policy by 0.08 μg/m^3^ per week, then this trend flattened in the post-policy period to 0.03 μg/m^3^ (95% CI 0.00, 0.06). An immediate change in concentrations (decline of 2.68 μg/m^3^) at the time of the policy is also evident. The slope difference coefficient is statistically significant for both contaminants, therefore the hypothesis of no change in air pollutants associated with the speed limits policy is rejected.

**Table 2:** City level analysis: change in NO_2_ and PM_2.5_ concentrations associated with the speed limits policy.

	Estimate	p
NO_2_		
Pre-2015 trend⁎	0.00 (−0.02,0.01)	0.84
Immediate change – start of policy (β_2_)	0.22 (−0.69,1.13)	0.64
Post-2015 trend⁎	−0.04 (−0.05,-0.03)	<0.001
***Slope difference (β***_***3***_***)***⸸	−0.04 (−0.05,-0.03)	<0.001

PM_2.5_		
Pre-2015 trend⁎	0.08 (0.06,0.10)	<0.001
Immediate change – start of policy (β_2_)	−2.68 (−3.70,-1.65)	<0.001
Post-2015 trend⁎	0.03 (0.00,0.06)	0.08
***Slope difference (β***_***3***_***)***⸸	−0.05 (−0.07,-0.03)	<0.001

⁎ Weekly change.

⸸ β refers to$\;Y_{t}=\beta_{0}+\beta_{1}\left(T-T_{i}\right)+\beta_{2}X_{t}+\beta_{3}\;\left(T-T_{i}\right)X_{t.}$

The results of the sensitivity analysis changing the start date of the policy to June 2016 are presented in supplementary Table 1. There is no effect for NO_2_ and a smaller effect, consistent with the main findings, for PM_2.5_. This suggests that the effect of the policy was immediate (as soon as it was approved) without a lag time.

Table 3 shows the controlled interrupted time series results. Trends in contaminants in close-proximity and far-from-radars were almost parallel (difference in slope differences for NO_2_ 0.00 ppb 95 % CI -0.02,0.03; for PM_2.5_ μg/m^3^ 0.02 95 % CI -0.01,0.05). In other words, there does not seem to be a moderation effect when monitors were close to speed radars versus when they were far from speed radars.

**Table 3:** Controlled interrupted time series analysis comparing NO_2_ and PM_2.5_ concentrations in close-proximity vs far-from-radar monitors, before and after the speed limits policy.

	Estimate	p
NO_2_		
Pre-2015 trend in close-proximity (Jan 2014 - Dec 2015) ⁎	0.00 (−0.02,0.01)	0.71
Pre-2015 trend in far-from-radar (Jan 2014 -Dec 2015) ⁎	0.00 (−0.02,0.02)	0.96
Immediate change in close-proximity monitors	0.68 (−0.74,2.10)	0.35
Immediate change in far-from-radar monitors	−0.20 (−1.09,0.68)	0.65
***Difference in immediate changes, close* vs *far (β6)***♦	0.88 (−0.69,2.46)	0.27
Post-2015 trend in close-proximity (Jan 2016-Dec 2018) ⁎	−0.04 (−0.06,-0.02)	<0.001
Post-2015 trend in far-from-radar (Jan 2016-Dec 2018) ⁎	−0.04 (−0.06,-0.02)	<0.001
Slope difference in close-proximity	−0.04 (−0.05,-0.03)	<0.001
Slope difference in far-from-radar	−0.04 (−0.06,-0.02)	<0.001
***Difference in slope differences (β7)***♦	0.00 (−0.02,0.03)	0.84

PM_2.5_		
Pre-2015 trend in close-proximity (Jan 2014 -Dec 2015) ⁎	0.06 (0.04,0.09)	<0.001
Pre-2015 trend in far-from-radar (Jan 2014 -Dec 2015) ⁎	0.10 (0.07,0.12)	<0.001
Immediate change – in close-proximity monitors	−1.78 (−3.70,0.14)	0.07
Immediate change – in far-from-radar monitors	−3.36 (−4.02,-2.70)	<0.001
***Difference in immediate changes, close* vs *far (β6)***♦	1.59 (−0.40,3.57)	0.12
Post-2015 trend in close-proximity (Jan 2016-Dec 2018) ⁎	0.02 (−0.02,0.06)	0.27
Post-2015 trend in far-from-radar ⁎	0.03 (0.01,0.06)	0.02
Slope difference in close-proximity	−0.04 (−0.07,-0.02)	<0.001
Slope difference in far-from-radar	−0.06 (−0.08,-0.04)	<0.001
***Difference in slope differences (β7)***♦	0.02 (−0.01,0.05)	0.25

⁎ Weekly change.

♦ β Refers to $Y_{t}=\beta_{0}+\beta_{1}\left(T-T_{i}\right)+\beta_{2}X_{t}+\beta_{3}\left(T-T_{i}\right)X_{t}+\beta_{4}G+\beta_{5}G\left(T-T_{i}\right)+\beta_{6}GX_{t}+\beta_{7}GX_{t}\left(T-T_{i}\right)$

For NO_2_, the trends for both groups of monitors were very similar, flat in the pre-policy period, and declining in the post-policy period. No differences were observed between groups. For PM_2.5_, both groups of monitors showed similar trends as described in the city analysis (increasing pre-policy and flattening, post-policy).

## 4 Discussion

The speed limit policy in Mexico City was associated with a reduction in NO_2_ and PM_2.5_. We show a change in the weekly trend with more negative weekly changes post intervention (by 0.04 ppb for NO_2_ and by 0.05 μg/m^3^ for PM_2.5_) . We did not observe a moderation effect of speed radars. The trends for both pollutants in monitoring stations close-to-radars and far-from-radars were almost parallel and consistent with the city level analysis. These results presumably show the net effect of lower emissions from reduction of speeds in access-controlled roads and primary roads and increased emissions from reduced traffic flow due to speed limits.

The evidence regarding the effect of speed limits and speed enforcement devices is mixed, with a systematic review judging the evidence to be insufficient (Bigazzi and Rouleau, 2017). When we compare our results with individual studies using similar quasi-experimental methodologies (i.e. not modelling studies), our results are consistent with some that find improvement in air quality associated with changes to speed limits (Dijkema et al., 2008; van Benthem, 2015) but not with others that find either no association or worsening air pollution (Bel et al., 2014; Folgerø et al., 2020). These differences may be due to the heterogeneity of the ‘interventions’ studied. While most measure specific roads, and changes from high speeds (Bel and Rosell, 2013), ours measures a city-level policy. Most studies, including ours, use air quality data from monitoring stations, which are representative of ambient regional levels but do not reflect near- road exposures (Bigazzi and Rouleau, 2017). This makes analyses less precise and prone to measurement error. Further, the effects of traffic management strategies tend to be moderate, and measuring small impacts on air quality is challenging given seasonality and exogenous factors that also influence traffic emissions and air quality (Bigazzi and Rouleau, 2017). Not all studies accounted for seasonality patterns and time-varying confounders. The external validity of studies, including ours, may be limited since local circumstances such as the mix of diesel vs gas vehicles or driver norms and response to road safety policies may be variable across countries.

In this study, we were not able to see differences in pollutant concentrations in the group of monitors in close proximity to monitors vs those further away. This may be due to limitations in the design of our study or may imply that speed radars do not modify traffic flows enough to cause higher emissions. We are unable to conclusively say which is the most plausible explanation since we do not have measured local data on acceleration and deceleration or of trip lengths before and after the policy. The buffer around monitoring stations that we used may have been too large to capture changes in emissions. Land use regression (LUR) models suggest that emissions from mobile sources have a greater impact on the concentration in the first 800 m of a high-traffic road. We tested different size buffers but made the pragmatic decision to keep 3.5 km buffers to better classify stations since smaller buffers led to very few monitors in ‘close-proximity’ to speed radars.

The findings of this study are relevant to public health since traffic-related air pollution (TRAP) is one of the major contributors to urban air pollution (HEI Panel on the Health Effects of Traffic-Related Air Pollution, 2010; Son et al., 2018). Epidemiological evidence shows that human exposure to TRAP increases the risk of a range of adverse health outcomes, such as lung cancer and cardiovascular and respiratory diseases (HEI Panel on the Health Effects of Traffic-Related Air Pollution, 2010). The speed limit policy appears to have had an unexpected positive effect on air pollution, contrary to what some environmental groups had claimed (Mochan, 2016; Olivares Alonso, 2016). The policy was also effective to reduce traffic deaths in the city (Quintero Valverde et al., 2022). However, it was not popular with drivers and was partially struck off by the new government that came into office in January 2019. If the findings of our study are confirmed, the additional environmental benefits of reducing speed limits in cities could provide further incentives to drivers of complying with speed limits and justifying enforcement. An experimental study which tested prevention messages highlighting the dual role of speeding in crash risk and air pollution were more effective at leading drivers to observe speed limits than only focusing on crash risk (Delhomme et al., 2010).

Our study has strengths and limitations. We use measured data from the SINAICA system for outcomes and covariates and a robust quasi-experimental methodology to estimate the change in outcomes at a particular point in time (Lopez Bernal et al., 2018a). As far as we are aware, there were no concurrent changes to policies or regulations that could affect air pollution other than the traffic regulations that we studied. The time series is long enough for both the pre- and post-policy periods (Lopez Bernal et al., 2018b). This is in contrast with a large proportion of the evidence on the association between speed and emissions and air pollution which arises from modelling studies (Baldasano et al., 2010; Bigazzi and Rouleau, 2017; Yang et al., 2021).

Our study also has limitations. We evaluated a city-wide policy, that affected a number of roads with lower speed limits, but we were unable to analyze changes in air pollution on specific roads because the air quality monitors measure air pollution of a large catchment area. A second limitation is that we do not have detailed data on traffic dynamics i.e. traffic flow and speed variation in the city over the study period. Specifically, this information is unavailable for the pre-policy period (Agencia Digital de Innovación Pública, 2021). To partially address this limitation, we adjusted for the number of registered vehicles, assuming correlation between number of registered vehicles and traffic delays. We believe this assumption is justified since there is some evidence that suggests increasing congestion in the city over time (Tomtom, 2022). Next, it was beyond our scope to analyze differential effects by area-level socioeconomic position. This may be of interest for future studies. Finally, quasi-experimental methodologies are not without limitations, specifically there could be unmeasured time-varying confounding factors. Further, having a control group for the city level analysis was not feasible since other similar cities did not implement speed limit policies during the study period nor have robust air quality monitoring systems like Mexico City.

In conclusion, the speed limits policy implemented in Mexico City in 2015 was associated with unexpected improvements in air pollution. This evidence together with results from studies that show that the policy was effective at reducing traffic injuries and deaths suggests that speed limits could be promoted in cities as a double-duty policy for public health.

## CRediT authorship contribution statement

**Jose Luis Texcalac-Sangrador:** Writing – review & editing, Writing – original draft, Methodology, Formal analysis, Data curation. **Carolina Pérez-Ferrer:** Writing – review & editing, Writing – original draft, Supervision, Project administration, Methodology, Funding acquisition, Formal analysis, Conceptualization. **Carolina Quintero:** Writing – review & editing, Supervision, Project administration, Data curation. **Francisco-Javier Prado Galbarro:** Writing – review & editing, Methodology, Formal analysis, Data curation. **Goro Yamada:** Writing – review & editing, Supervision, Methodology, Formal analysis. **Nelson Gouveia:** Writing – review & editing, Validation, Methodology, Conceptualization. **Tonatiuh Barrientos-Gutierrez:** Writing – review & editing, Supervision, Project administration, Methodology, Funding acquisition, Conceptualization.

## Declaration of competing interest

The authors declare that they have no known competing financial interests or personal relationships that could have appeared to influence the work reported in this paper.

## Data Availability

Data will be made available on request.

## Appendix A Supplementary data

Supplementary material Image 1
