# Supplementary material for: Speed limits and their effect on air pollution in Mexico City: A quasi-experimental study
Source: Sci Total Environ. 2024 May 10;924:171506. doi: 10.1016/j.scitotenv.2024.171506 (PMC10999787; doi:10.1016/j.scitotenv.2024.171506)
Supplement: Supplementary file 1 — Supplementary material [file mmc1.docx]

# Supporting information

Supplementary figure 1: Available daily mean of non-peak hours for PM_2.5_


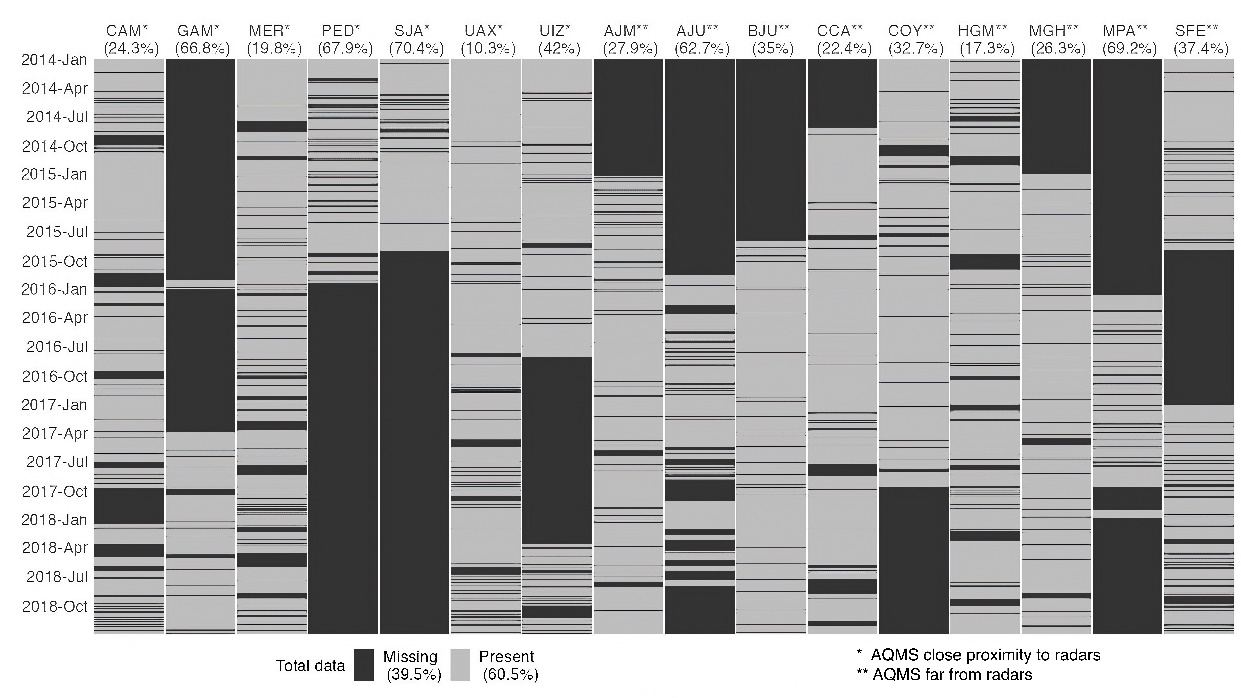


Supplementary figure 2. Available daily mean of non-peak hours for NO_2_


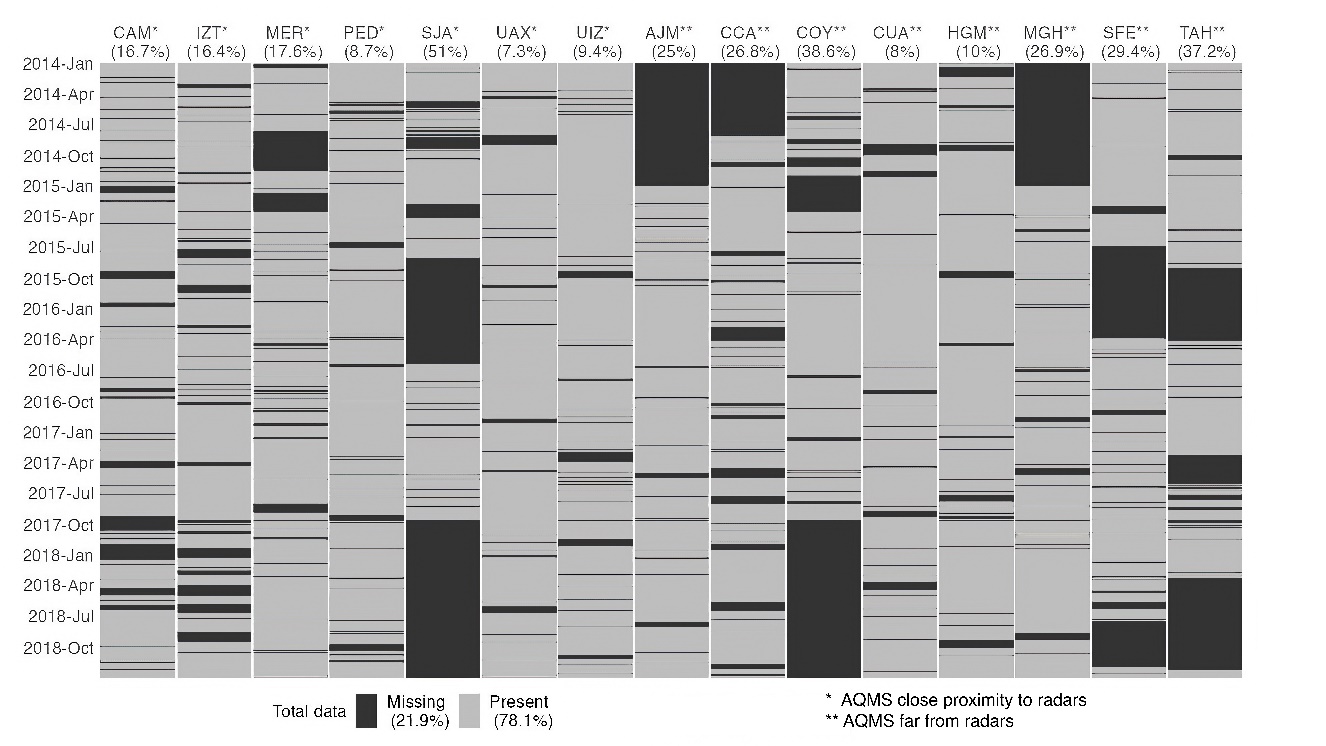


Supplementary figure 3. Adjustment for seasonality PM_2.5_


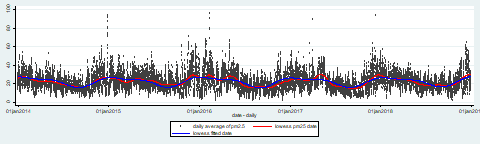


Supplementary figure 4. Adjustment for seasonality NO_2_


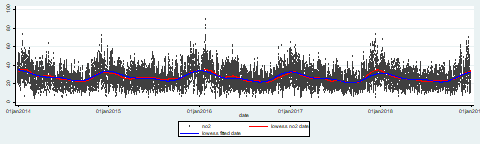


Supplmentary table 1: Sensitivity analysis changing start date of policy to June 15, 2016

|  | Estimate | p |
| --- | --- | --- |
| **NO_2_** |  |  |
| Level change – start of policy (β_2_) | -4.13 (-4.72,-3.54) | 0.000 |
| Pre-2015 trend * | 0.08 (0.06,0.11) | 0.000 |
| Post-2015 trend * | 0.08 (0.04,0.11) | 0.000 |
| ***Slope difference (β_3_)^⸸^*** | -0.01 (-0.03,0.01) | 0.494 |
| **PM_2.5_** |  |  |
| Level change – start of policy (β_2_) | -2.14 (-3.00,-1.29) | 0.000 |
| Pre-2015 trend * | 0.02 (0.00,0.03) | 0.020 |
| Post-2015 trend * | -0.01 (-0.03,0.01) | 0.199 |
| ***Slope difference (β_3_)^⸸^*** | -0.03 (-0.04,-0.02) | 0.000 |
